# Evaluation of an Infertile, All-Male ZZ Line Exhibiting Female-like Growth in Chinese Tongue Sole (*Cynoglossus semilaevis*): Growth Performance, Flesh Quality, and Muscle Metabolome

**DOI:** 10.3390/biology15010093

**Published:** 2026-01-01

**Authors:** Zhangfan Chen, Yinqi Wu, Lanqing Ding, Pengfei Li, Mengqi Wang, Xu Yan, Fangzhou Cheng, He Jiang, Zhongkai Cui, Songlin Chen

**Affiliations:** 1 State Key Laboratory of Mariculture Biobreeding and Sustainable Goods, Yellow Sea Fisheries Research Institute, Chinese Academy of Fishery Sciences, Qingdao 266071, China; chenzf@ysfri.ac.cn (Z.C.); wuyinqi0414ly@gmail.com (Y.W.); 18710713027@163.com (L.D.); fly2yongchuangshijie@gmail.com (P.L.); btswangmengqi@163.com (M.W.); yanxu_0210@foxmail.com (X.Y.); chengfangzhou0338@163.com (F.C.); jhgomora@163.com (H.J.); 2 Laboratory for Marine Fisheries Science and Food Production Processes, Qingdao Marine Science and Technology Center, Qingdao 266237, China; 3 School of Fisheries and Life Science, Shanghai Ocean University, Shanghai 201306, China; 4 College of Life Science, Qingdao University, Qingdao 266071, China

**Keywords:** Chinese tongue sole, *dmrt1*, genome editing, growth performance, sterile

## Abstract

Chinese tongue sole (*Cynoglossus semilaevis*) is an important marine aquaculture species that exhibits pronounced female-biased sexual size dimorphism, which limits the economic value of male stocks. The F4 generation of genome-edited *dmrt1*^−/−^ ZZ males carried a stable 8 bp deletion in *dmrt1*, developed ovarian lamella-like gonads and were completely sterile, as in vitro fertilization with their gonads produced no viable embryos. From 12 to 15 months post-hatch (mph), their growth rate was significantly higher than that of wild-type males and ultimately comparable to that of females. They weighed 3.2-fold heavier and measured 1.38-fold longer than their 15 mph wild-type male counterparts. Muscle nutritional composition and most texture traits of *dmrt1*^−/−^ ZZ males did not differ from wild-type females. However, their fat content was significantly lower than that of wild-type males, while their Omega6 content was significantly higher. Metabolomic analysis identified 1262 metabolites and revealed differential enrichment of pathways related to amino acids, energy, and antioxidant and neuromuscular metabolism, without evidence of detrimental shifts. Overall, *dmrt1* editing yields a fast-growing, functionally sterile male line whose flesh quality is similar to that of wild-type tongue sole, supporting its potential use in sex-control breeding in Chinese tongue sole and providing a valuable model for studying sexual size dimorphism.

## Simple Summary

Chinese tongue sole is a valuable farmed fish in which females grow much larger than males, reducing the profitability of male stocks. To address this, we use genome editing to disrupt the sex-determining gene *dmrt1* in male tongue sole. The edited fish grew as large as females and were completely unable to reproduce. Their muscle nutritional composition and texture were broadly comparable to those of wild-type fish, and no major abnormalities were detected in the measured parameters. These results demonstrated that *dmrt1* editing can create a fast-growing, infertile all-male population, offering a useful model for biological research.

## 1. Introduction

Chinese tongue sole (*Cynoglossus semilaevis*) is popularly distributed in northeast Asia. Due to its tender and juicy meat, Chinese tongue sole has high economic value, becoming one of the most important maricultural species in China [1]. Over the past twenty years, large-scale breeding technology for Chinese tongue sole has been successfully developed, establishing it as an excellent model for industrial aquaculture. However, females grow 2–4 times faster than males, resulting in smaller adult males in actual aquaculture, which lowers economic returns.

Through fine mapping of the whole genome, Doublesex and Mab-3-related transcription factor 1 (*dmrt1*) has been identified as a prime candidate crucial to male determination in Chinese tongue sole [1]. In mammals, *dmrt1* is a conserved transcription factor required for male gonadal differentiation [2]. Its paralog in medaka (*Oryzias latipes*), known as *dmy*, is a master sex-determining gene essential for testicular development [3]. Meanwhile, *dmrt1* has been suggested as a male specific marker in Siamese fighting fish (*Betta splendens*) [4], yellow drum (*Nibea albiflora*) [5], African scat (*Scatophagus tetracanthus*) [6], and Nile tilapia (*Oreochromis niloticus*) [7]. In other teleost species, *dmrt1* shows male-biased expression during sex differentiation and testicular maturation, such as mandarin fish (*Siniperca chuatsi*) [8], cobia (*Rachycentron canadum*) [9], Pacific bluefin tuna (*Thunnus orientalis*) [10], spotted knifejaw (*Oplegnathus punctatus*) [11], and leopard coral grouper (*Plectropomus leopardus*) [12].

To further investigate the function of *dmrt1* gene in Chinese tongue sole, we established an efficient genome editing method for flatfish embryos by using TALEN-mediated knockout for the first time, and successfully generated *dmrt1* mutant individuals of the F0 generation [13]. The *dmrt1*^+/−^ ZZ mutants displayed feminized characteristics, including sex-reverted gonads devoid of spermatids or sperm, as well as enhanced growth rates and higher body weight compared to wild-type males. Based on these findings, we propose that targeted knockout of *dmrt1* could be utilized to generate novel fast-growing strains of Chinese tongue sole through genome editing thereby addressing the issue of growth retardation in males. Importantly, the development of such a sterile, all-male strain also addresses key ethical and regulatory considerations. These sterile male fish might provide a strategy for controlling invasive fish species in natural habitats [14]. Furthermore, the comprehensive phenotypic and metabolic data generated in this study provide valuable baseline information that may inform future assessments of genome-edited fish in aquaculture-related contexts.

In this study, we generated the F4 generation of *dmrt1*^−/−^ ZZ mutants. According to our previous findings, their growth characters at 15 mph and their infertility were regarded as the primary outcomes. Their flesh quality was compared with wild-type tongues. Moreover, a muscle metabolomics investigation was carried out to explore the influence of *dmrt1* on metabolic pathways. This work provided a valuable fast-growing strain for the Chinese tongue sole aquaculture industry, and offered a potential model for probing the molecular basis underlying sexual size dimorphism in teleost fish.

## 2. Materials and Methods

### 2.1. Experimental Fish

**Figure 1:**
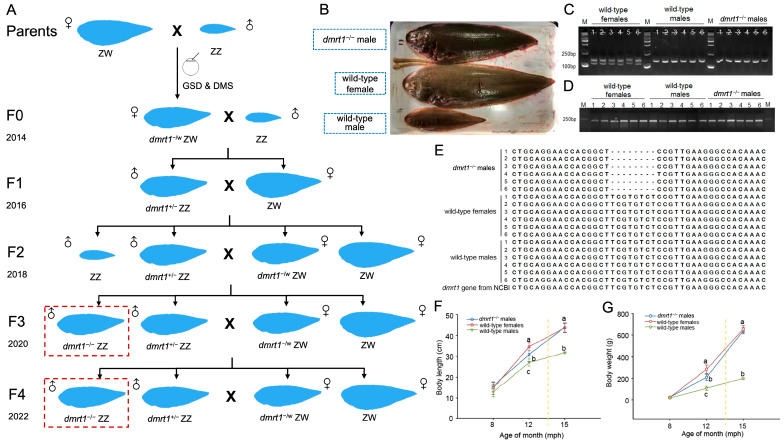
The pipeline for generating fast-growing Chinese tongue sole (*C. semilaevis*). (**A**) The schematic diagram for constructing the new strain with fast growing. The red-dashed boxes indicated the *dmrt1* homozygous mutant male fish. (**B**) The morphological alternations of *dmrt1^−/−^* male compared with the wild-type female and male at 15 mph. (**C**) The identification of genetic sex in *dmrt1^−/−^* and wild-type tongue soles. Each group contained 6 individual fish (labeled from 1 to 6). Females exhibited two bands (one in 134 bp and the other in 169 bp), while males exhibited only one band in 169 bp. (**D**) The amplified *dmrt1* fragments in *dmrt1^−/−^* and wild-type tongue soles. (**E**) Sequence alignment of *dmrt1* amplification fragments between *dmrt1^−/−^* and wild-type tongue soles. *dmrt1^−/−^* males shows an 8 bp deletion compared to the wild-type sequence. Dashes represent alignment gaps introduced to optimize sequence matching. *Dmrt1* gene sequence from NCBI serves as the reference. Comparisons of body lengths (**F**) and body weights (**G**) among *dmrt1^−/−^* males, wild-type males, and females at 8 mph, 12 mph, and 15 mph. The different letters in F and G indicate significant differences (*p* < 0.05).

All fishes used in this study were cultured in Tangshan Weizhuo aquaculture Co., Ltd. (Tangshan, China). To induce spawning, mature broodstocks were injected with luteinizing hormone-releasing hormone (LHRH-A30.4, 4 μg/kg body weight) and domperidone (DOM, 1–2 mg/kg body weight) [13]. For the F0 generation, we generated *dmrt1* knockout tongue sole by using TALEN-mediated genome editing following the protocol described by Cui et al. [13]. Subsequently, all F0 individuals were subjected to genetic sex determination (GSD) and *dmrt1*-mutation screening (DMS). DNA was extracted with TIANamp Marine Animal DNA kit (TIANGEN, Beijing, China). PCR amplification of GSD was performed using the sex-F/R primers (sex-F: 5′-CCTAAATGATGGATGTAGATTCTGTC3′, sex-R: 5′-GATCCAGAGAAAATAAACCCAGG-3′), and genetic sex was determined based on gel electrophoresis results. The *dmrt1* fragment containing mutant region was amplified using the *dmrt1*-F/R primers (*dmrt1*-F: 5′-CGGGCAAAGGGAGAAGG-3′ and *dmrt1*-R: 5′-AAAAACATCTCCTGAGGGCTAA-3′) and sequenced by Tsingke (Beijing, China). All the PCR assays were performed following the procedures mentioned in Cui et al. [13]. The breeding scheme for generating F1 through F4 generations is illustrated in Figure 1A. For each subsequent generation, broodstock selection was based on combined GSD and DMS results. To assess potential off-target effects, genomic DNA was extracted from ten fish: three wild-type females, three wild-type males, and four *dmrt1*^−/−^ ZZ males. Whole-genome resequencing was performed by Novogene Co., Ltd. (Beijing, China). Potential off-target loci were provided by Cui et al., [13]. Subsequently, the potential off-target sequence was identified by aligning the whole-genome resequencing data to the reference genome data of Chinese tongue sole.

Each concrete tank covered an area of 35 square meters. The stocking density decreased as the fish grew, with approximately 65 fish per square meter at 8 months post-hatch (mph), 30 fish per square meter at 12 mph, and 15 fish per square meter at 15 mph. The water temperature was maintained at 23–24 °C, with a salinity of 18–22‰. The photoperiod involved light exposure only during feeding and water discharge periods, totaling 3 h per day. Fish were fed a commercial diet twice daily (specifications: crude protein ≥ 52.0%, crude fat ≥ 9.0%, crude fiber ≤ 2.0%, crude ash ≤ 16.0%, lysine ≥ 2.5%). Fish were fasted for 24 h prior to experimentation or sampling. The *dmrt1*^−/−^ males and wild-type tongue soles were co-cultured in a pond at a 1:1:1 ratio, with a total of three such ponds set up at 8 mph. As the fish grew and stocking density decreased, they were redistributed into additional tanks. For each sampling event, healthy fish (free of skin ulcers, ascites, and skeletal deformities) were randomly selected from all available tanks and grouped using GSD and DMS. If the measurements of body weight and full length were required, the fish were returned to the rearing tank subsequent to fin clip sampling and data collection. If tissue sampling was required, MS222 (Sigma-Aldrich, Oakville, ON, Canada) was used for anesthesia to minimize fish suffering (solubilized in seawater, final concentration 20 mg/L, fish was treated for 5 min) during experimental procedure [15].

### 2.2. Growth and Reproduction Performance Analysis

The full lengths and body weights were recorded from *dmrt1*^−/−^ ZZ males and wild-type tongue soles at 8 mph, 12 mph, and 15 mph. The age at sampling was tightly controlled, with all samples collected within a one-week tolerance window (±1 week) of each target month. Thirty individuals were sampled for each group in each tank at one time point. Total body length was measured by a standardized ruler. The body weight was determined with an electronic scale.

To compare reproduction performance between *dmrt1*^−/−^ ZZ males and wild-type males, the eggs obtained through artificial induction from one wild-type female tongue sole were divided into two groups. One group was fertilized with sperm from wild-type males, and the other with sperm from *dmrt1*^−/−^ ZZ males. A single wild-type female can produce approximately 150 mL of eggs, with each milliliter containing roughly 1000 eggs. We harvested eggs from five wild-type females to repeat this experiment five times. Since the homozygous males were unable to release sperm by stripping [13], their testes were dissected, minced, and mixed with the eggs for attempted in vitro fertilization. As the fertilized eggs of Chinese tongue sole are buoyant, those that sank to the bottom were considered non-viable. Floating eggs were collected at 10 and 24 h post-fertilization (hpf) for the observation of embryonic development stages.

### 2.3. The Evaluation of dmrt1 mRNA Expression Level in C. semilaevis Tissues

Seven tissues, including heart, liver, gonad, intestine, muscle, skin, and blood were collected from females and males, as well as *dmrt1^−/−^* ZZ males of 12 mph. Total RNA was extracted from collected samples by using TRIzol reagent (Invitrogen, Carlsbad, CA, USA). Genomic DNA removal and first strand cDNA was synthesized by using ReverTra Ace qPCR RT Master Mix (Toyobo, Osaka, Japan). As for RT-PCR analysis, a pair of gene specific primers (*dmrt1*-RTF: 5′-CCGGACGGCTTCGTGTC-3′ and *dmrt1*-RTR: 5′-CTTCCACAGGGAGCAGGCAGT-3′) were designed to span the TALEN-targeted region [12]. Because the primers annealed within the knockout site, their binding was disrupted when the target sequence was deleted. As a result, these primers should fail to produce an amplification product in *dmrt1^−/−^* ZZ males. *β-actin* was enrolled as the internal control (amplified by *β*-*actin*-RTF: GCTGTGCTGTCCCTGTA and *β*-*actin*-RTR: GAGTAGCCACGCTCTGTC). The RT-PCR program was set as follows: 94 °C for 5 min, 94 °C for 30 s, 58 °C for 30 s, 72 °C for 30 s and 30 cycles, and 72 °C for 5 min. The PCR products were analyzed with agarose gel electrophoresis.

### 2.4. Western Blot

**Figure 2:**
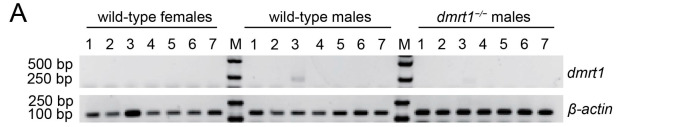

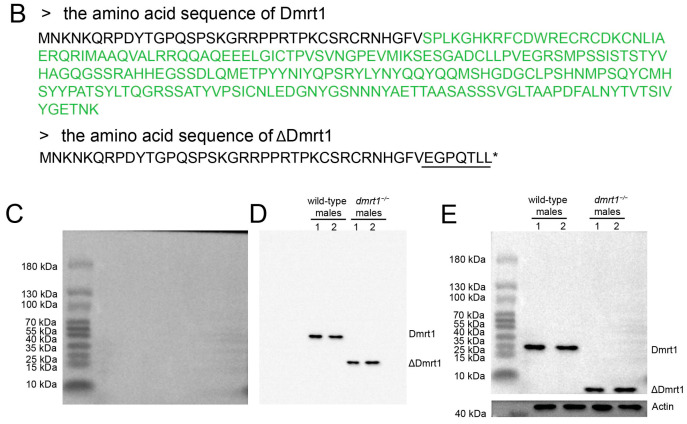

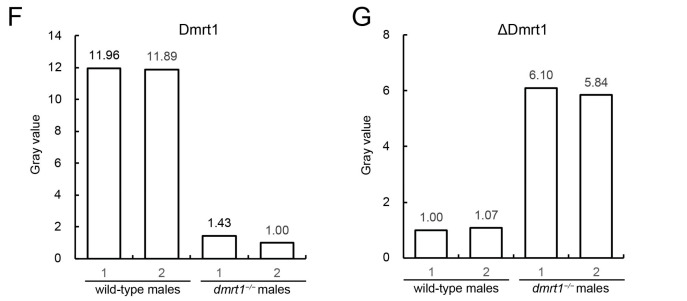
*dmrt1* expression at both mRNA level and protein level. (**A**) RT-PCR analysis of *dmrt1* expression in several tissues from wild-type females, wild-type males, and *dmrt1^−/−^* males. Tissues include heart (1), liver (2), gonad (3), intestine (4), muscle (5), skin (6), and blood (7). Raw data of this figure could be found in Figure S4. (**B**) The amino acid sequences of Dmrt1 and ∆Dmrt1. The underlined amino acids represented the amino acid following the mutation site, and the asterisk indicated the termination of the peptide. The amino acid sequences of Dmrt1 in green and ∆Dmrt1 were used as antigens for antibody preparation. (**C**–**G**) Western blot membrane of Dmrt1 (~28 kDa), ∆Dmrt1 (~5 kDa), and Actin (~43 kDa) protein detected with anti-Dmrt1 (homemade; 1:1000) and anti-Actin (homemade, 1:1000) antibodies, respectively. Actin was used as the internal control. We first captured a white-light image of the transferred membrane to document the pre-stained protein marker (**C**). Afterward, the membrane was incubated with ECL substrate, and the chemiluminescent signal was recorded using the gel imaging system (**D**). Finally, the white-light image and the ECL chemiluminescence image were merged to generate the final composite figure (**E**). The gray values of Dmrt1 protein bands (**F**) and ∆Dmrt1 protein bands (**G**) were analyzed based on the chemiluminescent signals.

The coding sequence of Dmrt1, corresponding to the protein region highlighted in green in Figure 2B, was synthesized by General Biol Company (Chuzhou, China) and cloned into the pET-32a expression vector. The recombinant His-tagged Dmrt1 protein was expressed in *Escherichia coli* and purified using Ni-NTA affinity chromatography. To enhance immunogenicity, a triple repeat of the truncated Dmrt1 peptide sequence (ΔDmrt1 in Figure 2B) was designed and a His tag was added to the N-terminus for purification and detection. The recombinant peptide was chemically synthesized by General Biol Company (China). The purified His-tagged Dmrt1 proteins (purity ≥ 85%) and the synthesized ΔDmrt1 peptide were used as antigens for rabbit immunization, respectively. After four rounds of immunization, the antibody titer was confirmed to be no less than 1:10,000, and antibodies were purified via exsanguination.

Proteins were extracted from the frozen gonad tissues by using RIPA buffer (Beyotime, Shanghai, China). After polyacrylamide gel electrophoresis, proteins were transferred onto a PVDF membrane. The membrane was blocked with 5% BSA and, respectively, incubated with homemade Dmrt1 antibody and ΔDmrt1 antibody at 4 °C overnight (1:1000 dilution). Then, it was rinsed with PBST and incubated with goat anti-rabbit IgG-HRP (1:5000 dilution). Positive signal was developed by using ECL chemiluminescence kit (Solarbio, Beijing, China).

### 2.5. Histological Analysis

Tissues including gonads, liver, spleen, heart, kidney, and muscle were carefully dissected from wild-type males and females, and *dmrt1^−/−^* ZZ males of 18 mph. The tissues were fixed in 4% paraformaldehyde in phosphate-buffered saline (PBS, pH 7.4), dehydrated in serial dilutions of ethanol, embedded in paraffin wax, and cut into 7 µm-thick sections. The sectioned slides were then stained by using hematoxylin and eosin (H&E).

### 2.6. Flesh Quality Evaluation

Muscle tissues were carefully dissected from the bones of multiple individuals within each group (labeled as 12 mph). As the muscle mass from a single fish was insufficient for the minimum analytical requirement, we pooled tissues from two fish to form one composite sample. Each experimental group consisted of three such replicate samples. Samples were stored at −80 °C and shipped on dry ice to SGS-CSTC Standards Technical Service (Qingdao) CO., Ltd (Qingdao, China). Nutritional components including moisture, ash, crude protein and crude fat, amino acid components, and fatty acid components were measured in accordance with Chinese national standards.

Ten fish from wild-type females and *dmrt1^−/−^* ZZ males of tongue sole were used for the evaluation of flesh quality. The entire fish filet was excised from the backbone and shipped on dry ice to Standards Testing Group Co., Ltd. (Qingdao, China). From each fillet, six muscle blocks (three from each side of the lateral line) were collected. These blocks were then randomly divided into two groups: three for TPA (Texture Profile Analysis) testing and three for shear force measurement. A section of muscle (2 cm^3^) from each specimen was excised and placed on the platform of a TMS-Pro texture meter (Food Technology Corporation, Sterling, VA, USA). A cylindrical metal probe with a diameter of 36 mm was used for the TPA test. Each sample underwent 2 cycles of compression analysis with a compression level of 60%, with a 30 mm/min test speed and 0.05 N trigger stress. For the shear force measurement, a precision cutting blade was employed. The test commenced (trigger force: 0.05 N) once the probe contacted the sample, shearing downward at 30 mm/min until complete severance.

### 2.7. Metabolomics Analysis

Muscle tissues were dissected from 10 individuals of wild-type males, 10 of wild-type females, and 6 of *dmrt1^−/−^* ZZ males, then flash-frozen and homogenized in liquid nitrogen. The powder was thoroughly resuspended in chilled ethanol–acetonitrile–water (2:2:1, *v*/*v*), and then set aside at −20 °C for 10 min. After centrifugation at 14,000× *g* for 20 min, the supernatant was vacuum dried and reconstituted in acetonitrile–water solvent (1:1, *v*/*v*). The solution was centrifuged at 14,000× *g* for 15 min and the resulting supernatant was analyzed using a Vanquish UHPLC system (Thermo Fisher Scientific, Dreieich, Germany) coupled to an Orbitrap Exploris™ 480 mass spectrometer (Thermo Fisher Scientific, Dreieich, Germany) in Shanghai Applied Protein Technology Co., Ltd. (Shanghai, China).

Separation was performed on a 2.1 mm × 100 mm ACQUIY UPLC BEH Amide 1.7 μm column (Waters, Milford, MA, USA). The mobile phase consisted of 25 mM ammonium acetate and 25 mM ammonium hydroxide in water (solvent A) and acetonitrile (solvent B). The gradient elution program was as follows: 95% B held for 0.5 min, linearly decreased to 65% over 6.5 min, further decreased to 40% B over 1 min and held for 1 min, then increased to 95% B in 0.1 min, followed by a 2.9 min re-equilibration period.

After raw data conversion with ProteoWizard MSConvert, peak identification, integration, peak alignment, and normalization were performed using XCMS software (version 3.2). The processed data were then analyzed using the R package (ropls: 1.18.8) for Pareto-scaled principal component analysis (PCA) and orthogonal partial least-squares discriminant analysis (OPLS-DA). A 200-permutation test was performed by repeatedly constructing OPLS-DA models using the metabolite data with permuted labels, and the Q^2^_permuted value was recorded for each permutation. A 7-fold cross-validation procedure was applied. The OPLS-DA models were validated based on interpretation of variation in Y (R^2^Y) and the model’s predictive capacity (Q^2^) in cross-validation. Student’s t-test was utilized to identify significant differences in metabolite levels between two groups. Metabolites with a variable importance in the projection (VIP) > 1 and *p*-value < 0.05 were regarded as differentially accumulated metabolites (DAMs). It is noted that the *p*-values were not adjusted for multiple testing (e.g., by false discovery rate) in this exploratory analysis; therefore, the results should be interpreted as generating hypotheses for future validation. Metabolic pathway enrichment analysis was performed using the Kyoto Encyclopedia of Genes and Genomes (KEGG).

### 2.8. Statistical Analysis

The data obtained was analyzed using Statistical Product and Service Solution (SPSS) software (Version 20.0, IBM, Chicago, IL, USA). Mann–Whitney test was used to test flesh texture between wild-type females and *dmrt1^−/−^* ZZ males, followed by Holm–Bonferroni correction. Meanwhile, one-way analysis of variance (ANOVA) along with Tukey’s post hoc tests was employed for comparisons involving more than two sets of data, such as growth indicators and flesh nutritional analysis. Levene’s test was performed for homogeneity of variances and calculation of partial eta squared (η^2^) was calculated as the effect size. All data were expressed as mean ± standard deviation. Values were considered as significant at *p* < 0.05.

## 3. Results

### 3.1. F4 dmrt1^−/−^ ZZ Males Possessing a Growth Rate Comparable to Females

At 15 mph, the F4 *dmrt1^−/−^* ZZ male was significantly larger than the wild-type male, with a body size closely resembling that of the wild-type female (Figure 1B). As only 25% of the offspring from F3 parents were theoretically expected to be *dmrt1^−/−^* ZZ males, it remained necessary to verify their genetic sex and *dmrt1* genotype. Agarose gel electrophoresis revealed that F4 *dmrt1^−/−^* ZZ males exhibited a single band at 169 bp, confirming their genetic male identity (Figure 1C). Sequencing of the *dmrt1* fragment amplified across the mutant region (Figure 1D) detected an 8 bp-deletion in F4 *dmrt1^−/−^* ZZ males (Figure 1E). The results showed that the only sequence matching a predicted off-target site corresponded to the intended *dmrt1* locus on the Z chromosome (position 11740124–11761213) (Supplementary Table S1 and Supplementary Figure S1), indicating that no off-target editing was detected in the *dmrt1*^−/−^ ZZ males.

Regarding growth performance, F4 *dmrt1^-/-^* ZZ males exhibited a significantly higher growth rate than wild-type males (Figure 1F,G). No significant differences in body weight or body length were observed between them and wild-type fish at 8 mph. Thereafter, their growth rate accelerated, resulting in a 1.96-fold increase in body weight and a 1.14-fold increase in body length at 12 mph compared to wild-type males (body weight: η^2^ = 0.871, *p* < 0.05, 95% CI [57.12, 148.68]; body length: η^2^ = 0.670, *p* < 0.05, 95% CI [0.25, 7.28]). However, they remained significantly lighter and shorter than wild-type females (body weight: η^2^ = 0.871, *p* < 0.05, 95% CI [−119.87, −28.30]; body length: η^2^ = 0.670, *p* < 0.05, 95% CI [−7.21, −0.19]). By 15 mph, their average body weight (638.76 ± 27.63 g) and average body length (43.8 ± 2.39 cm) were comparable to those of wild-type females (654.53 ± 32.30 g; 43.5 ± 2.29 cm). Meanwhile, they were 3.2 times heavier and 1.38 times longer than 15 mph wild-type males, showing significant differences (body weight: η^2^ = 0.988, *p* < 0.05, 95% CI [385.81, 492.98]; body length: η^2^ = 0.903, *p* < 0.05, 95% CI [7.80, 16.33]).

### 3.2. Reproduction Performance Analysis

**Figure 3:**
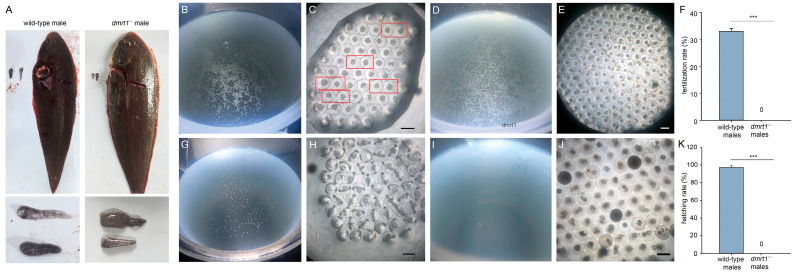
A comparison of reproductive performance between wild-type and *dmrt1^−/−^* males revealed significant differences. The phenotype comparison between wild-type male’s testis and *dmrt1^−/−^* male’s gonad (**A**). At 10 h post-fertilization (hpf), embryos fertilized by wild-type sperm developed normally (**B**), with some reaching the early gastrula stage and forming a germ ring (embryos in red frame in (**C**)). In contrast, eggs fertilized by *dmrt1^−/−^* sperm showed no signs of development (**D**,**E**). The percentage of embryos developing normally was significantly higher when fertilized by wild-type males than by *dmrt1^−/−^* males (**F**). By 24 hpf, embryos from wild-type males had developed to the somite stage (**G**,**H**), whereas eggs fertilized by *dmrt1^−/−^* males had sunk (**I**) and were non-viable (**J**). Furthermore, none of the eggs fertilized by *dmrt1^−/−^* males hatched (**K**). The scale bar = 1 mm. Three asterisks (***) indicates an extremely significant difference (*p* < 0.001).

The phenotype of wild-type males’ testis and *dmrt1^−/−^* males’ gonad were compared (Figure 3A). The testis of wild-type males appeared whitish on the surface due to the abundance of mature sperm, whereas the gonad of *dmrt1*^−/−^ male exhibited a grayish surface appearance. At 10 hpf, most eggs fertilized with wild-type sperm floated on the water surface (Figure 3B). Microscopic examination revealed that the embryos had developed to the early gastrula stage, with the formation of a germ ring (Figure 3C). In contrast, although a small proportion of eggs fertilized with sperm from *dmrt1^−/−^* ZZ males floated (Figure 3D), none exhibited normal embryonic development (Figure 3E). The percentage of embryos developing normally was significantly higher when fertilized by wild-type male ((33.33 ± 1.15)%) than by *dmrt1^−/−^* ZZ males (Figure 3F). By 24 hpf, embryos from wild-type sperm had reached the somite stage (Figure 3G and 3H) and their hatching rate was (96.67 ± 1.53) %. However, all eggs fertilized with sperm from *dmrt1^−/−^* ZZ males had sunk to the bottom (Figure 3I). Observation of these sunken eggs confirmed that they were non-viable (Figure 3J), resulting in a zero hatching rate (Figure 3K). In conclusion, the results indicate that male tongue sole with homozygous mutation of the *dmrt1* gene are completely sterile and unable to produce functional sperm.

### 3.3. dmrt1 Expression Levels in F4 dmrt1^−/−^ ZZ Males

As a sex determination gene located on the Z chromosome of the tongue sole, *dmrt1* expression was not detected in any tissues of wild-type females or *dmrt1^−/−^* ZZ males. In contrast, in wild-type males, *dmrt1* expression was exclusively observed in the testis (Figure 2A). Furthermore, Western blot analysis revealed a truncated Dmrt1 protein with a molecular weight of ~5 kDa in *dmrt1^−/−^* ZZ males (Figure 2E). Following gene editing of *dmrt1*, the modified base sequence generated a premature termination codon, leading to early termination of translation and thus producing the shortened protein.

### 3.4. Tissue Histology of F4 dmrt1^−/−^ ZZ Males and Wild-Type Fishes

**Figure 4:**
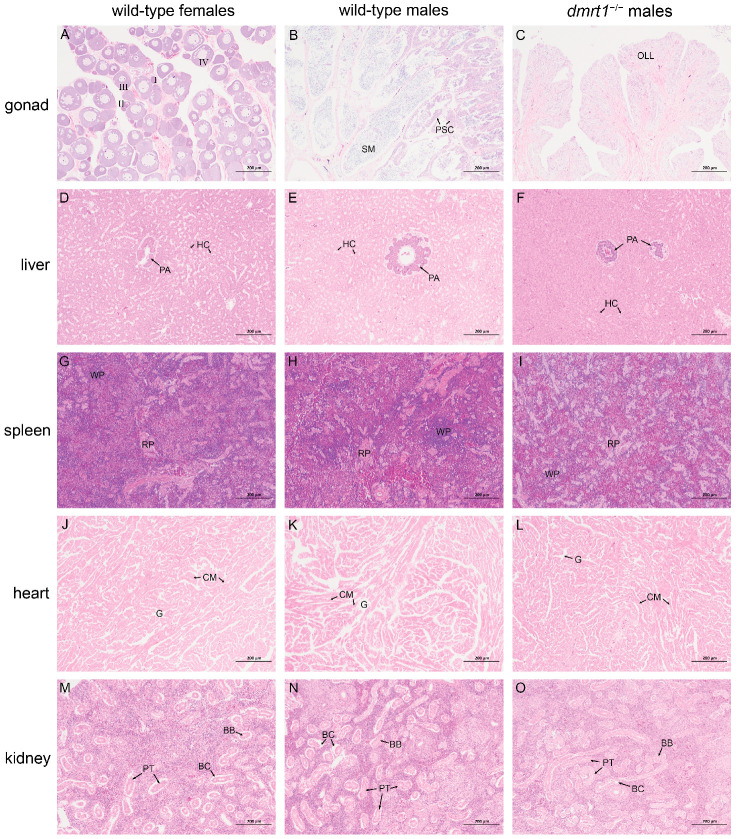
The histological morphology of tissues from 12 mph wild-type females, wild-type males, and *dmrt1*^−/−^ males of *C. semilaevis*, including gonads (**A**–**C**), liver (**D**–**F**), spleen (**G**–**I**), heart (**J**–**L**), and kidney (**M**–**O**). I, II, III, and IV suggest the different stages of oocytes. BB, brush border; BC, Bowman’s capsule; CM, cardiomyocyte; G, interstitial blood vessel; HC, hepatic cell; OLL, ovarian lamella-like; PA, pancreatic acina; PSC, primary spermatocytes; PT, proximal tubule; RP, red pulp; SM, sperm; WP, white pulp. The scale bar = 200 μm.

Unlike the ovaries in wild-type females or the testes in wild-type males (Figure 4A,B), the gonad of *dmrt1^−/−^* ZZ males exhibited neither seminiferous lobuli nor ovarian lamella, but contained some ovarian lamella-like tissues (Figure 4C). This phenotype was consistently observed in the *dmrt1* knockout from F0 generation onward [13]. Aside from gonads, no major abnormalities were detected in the other tissues examined, including liver, spleen, heart, and kidney (Figure 4D–O). In the liver of *dmrt1^−/−^* ZZ males, pancreatic acini could be observed, and numerous hepatocytes are tightly arranged to form hepatic cords (Figure 4F). Its spleen has an intact and clear structure, containing densely packed lymphocytes and blood cells (Figure 4I). The myocardial striations of *dmrt1^−/−^* ZZ males are distinct, with myocardial fibers arranged normally and no signs of collision and rupture. Intercellular gaps existed between the cardiomyocytes (Figure 4L). In its kidney, a large number of nephrons were distributed, with renal capsules and proximal convoluted tubules visible. The apical surface of the epithelial cells in the proximal convoluted tubules exhibited a well-developed brush border (Figure 4O).

### 3.5. Assessment of Nutritional Quality in F4 dmrt1^−/−^ ZZ Males

**Figure 5:**
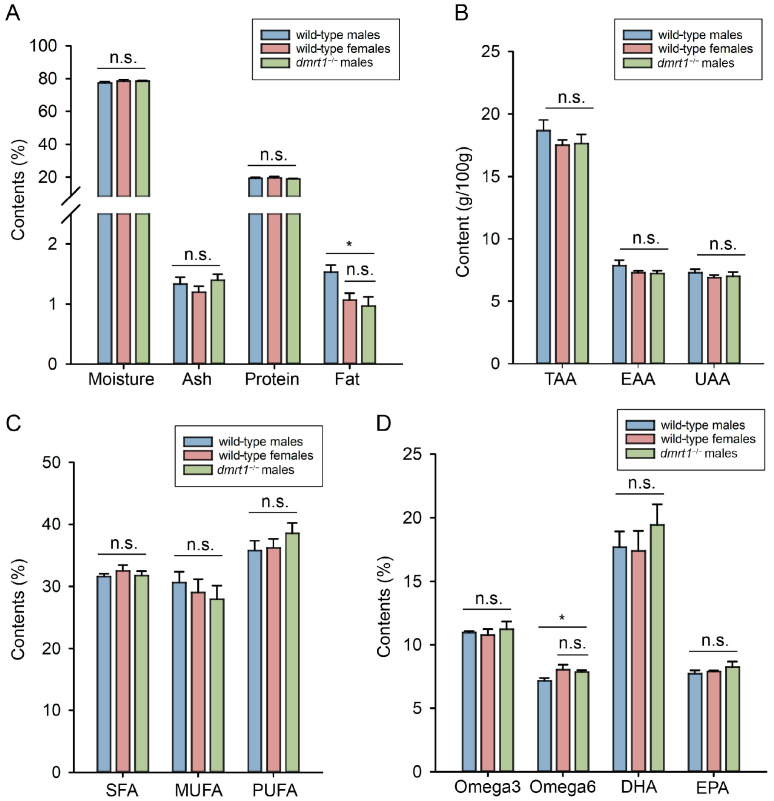
Evaluation of muscle nutrients in wild-type males, wild-type females, and *dmrt1*^−/−^ males of *C. semilaevis*, including nutritive compositions (**A**), amino acid components (**B**), and fatty acid components (**C**,**D**). Bars represent the mean ± SD (*n* = 3). An asterisk (*) indicates a significant difference (*p* < 0.05). n.s. means no significance. TAA, total amino acid; EAA, essential amino acid; UAA, umami amino acid; SFA, saturated fatty acid; MUFA, monounsaturated fatty acid; PUFA, polyunsaturated fatty acid; Omega3, omega3 fatty acid; Omega6, omega6 fatty acid; DHA, docosahexaenoic acid; EPA, eicosapentaenoic acid.

We assessed the nutrient composition of muscle tissue in 12 mph *dmrt1^−/−^* males by evaluating the proximate composition, amino acids, and fatty acids (Figure 5). A total of 16 amino acids and 24 fatty acids were identified and quantified, with detailed data provided in Supplementary Tables S2–S4. As for all the nutrient components mentioned above, no significant difference existed between *dmrt1*^−/−^ males and wild-type females. However, fat in muscles from wild-type males was significantly higher than *dmrt1^−/−^* males and wild-type females (η^2^ = 0.846, wild-type males vs. wild-type females: *p* < 0.05, 95% CI [0.24, 0.89]; wild-type males vs. *dmrt1*^−/−^ males: *p* < 0.05, 95% CI [0.14, 0.79], Figure 5A), while wild-type males had significantly less Omega6 than fishes from the other two groups (η^2^ = 0.735, wild-type males vs. wild-type females: *p* < 0.05, 95% CI [−1.58, −0.18]; wild-type males vs. *dmrt1*^−/−^ males: *p* < 0.05, 95% CI [−1.4, −0.01], Figure 5D).

### 3.6. Muscle Structure and Texture of F4 dmrt1^−/−^ ZZ Males

**Figure 6:**
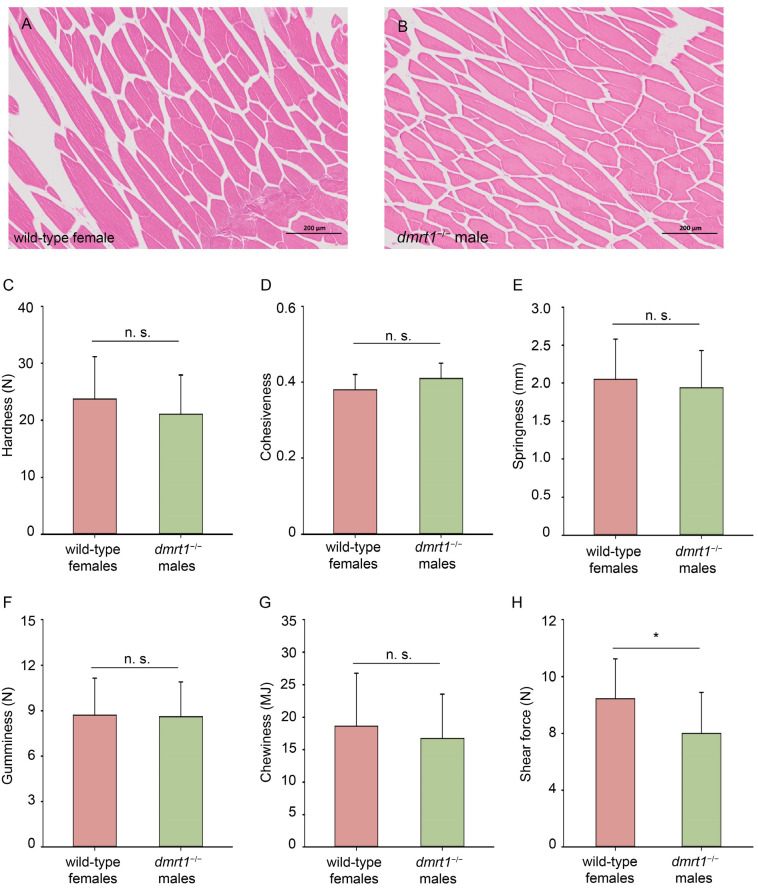
The structure and texture of muscle from wild-type females and *dmrt1*^−/−^ males. The histological analysis showed the cross-section diagram of the wild-type female (**A**) and the *dmrt1*^−/−^ male (**B**). Hardness (**C**), cohesiveness (**D**), Springiness (**E**), Gumminess (**F**), Chewiness (**G**), and Shear force (**H**) were examined for texture analysis. n.s., not significant; an asterisk (*) indicates a significant difference (*p* < 0.05).

The histological result showed that the cross-section diagram of muscles from the *dmrt1*^−/−^ male were similar to that from the wild-type female (Figure 6A,B). To compare the muscular structure, hardness, cohesiveness, springiness, gumminess, chewiness, and shear force were measured. Results showed that the shear force of wild-type females was 1.31-fold as much as those of *dmrt1*^−/−^ males (Effect size r = −0.42, *p* < 0.05, Figure 6H). In addition, the other parameters showed no significant difference between *dmrt1*^−/−^ males and wild-type females, including hardness, cohesiveness, springiness, gumminess, and chewiness (Figure 6C–G). PCA of muscle texture revealed that most samples from both groups clustered closely together with a considerable overlap of their 95% confidence ellipses (Supplementary Figure S2), indicating that the overall texture structures of the two groups shared a high degree of similarity, yet inter-group differences were not the primary source of data variation.

### 3.7. Metabolome Analysis

We performed pairwise comparisons among the three sample groups, primarily comparing *dmrt1^−/−^* ZZ males with wild-type females, and *dmrt1^−/−^* ZZ males with wild-type males. The OPLS-DA score plots showed a clear separation between each pair of groups (Supplementary Figure S3A–D). Model validity was assessed based on Q2, separation between R2Y and Q2, and permutation testing. After cross-validation, the OPLS-DA fit criteria listed in Supplementary Table S5 revealed that all the values of R^2^Y were less than 1 and Q^2^ greater than 0.4, indicating the stable and reliable model. The permutation test plots for the OPLS-DA models of both comparison groups showed that as the retention level in the permutation gradually decreased, the R^2^ and Q^2^ of the random models consistently declined. This indicated that the original model did not exhibit overfitting (Supplementary Figure S3E–H), suggesting acceptable predictive performance for exploratory analysis.

**Figure 7:**
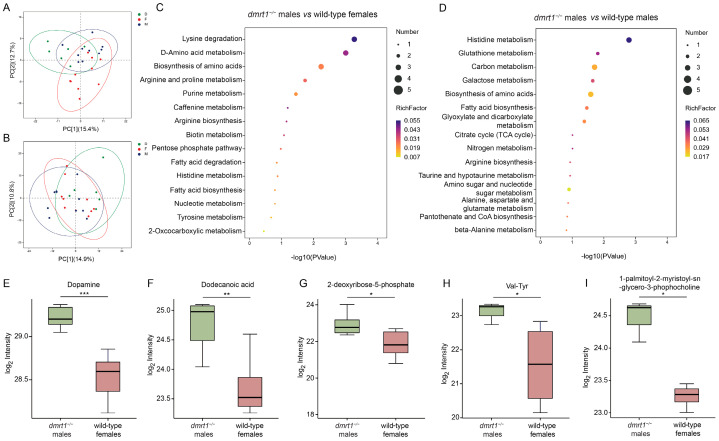

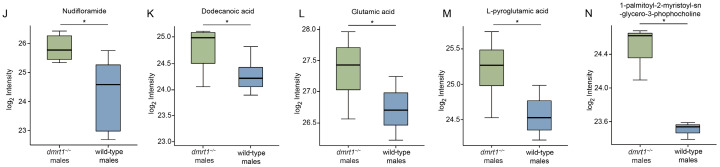
The metabolome analysis among *dmrt1^−/−^* males and wild-type females and males. The PCA of the metabolites in positive ion mode (**A**) and negative ion mode (**B**). The enriched pathways of metabolites in *dmrt1^−/−^* males compared to wild-type females (**C**) and wild-type males (**D**), respectively. Several metabolites were up-regulated in *dmrt1^−/−^* males (**E**–**N**). D, *dmrt1^−/−^* ZZ males; F, wild-type females; M, wild-type males; *, *p* < 0.05; **, *p* < 0.01; ***, *p* < 0.001.

A total of 1262 metabolites were identified in the muscle tissues of male, female, and *dmrt1^−/−^* ZZ male of *C. semilaevis* using both positive and negative ion modes (Figure 7A,B). Based on chemical taxonomy, organic acids and derivatives accounted for the largest proportion (23.46%); followed by lipids and lipid-like molecules (21.95%), and organoheterocyclic compounds (14.34%). According to the screening criteria (VIP > 1.0, FC > 1.5 or FC < 0.67, *p* < 0.05), 98 different metabolites were identified between *dmrt1^−/−^* ZZ males and wild-type females, containing 17 up-regulated and 81 down-regulated in *dmrt1^−/−^* ZZ males (Supplementary Tables S6 and S7). Compared with wild-type males, 108 metabolites showed significant differences in *dmrt1^−/−^* ZZ males, including 7 up-regulated and 101 down-regulated (Supplementary Tables S8 and S9). KEGG pathway analysis suggested that differential metabolites between *dmrt1^−/−^* ZZ males and wild-type females were mainly enriched in lysine degradation, D-amino acid metabolism, biosynthesis of amino acid, arginine and proline metabolism, and purine metabolism (Figure 7C). Meanwhile, differential metabolites between *dmrt1^−/−^* ZZ males and wild-type males were primarily enriched in histidine metabolism, glutathione metabolism, carbon metabolism, galactose metabolism, and biosynthesis of amino acid (Figure 7D). The up-regulated metabolites were associated with neurotransmission (e.g., dopamine, nudifloramide, and glutamic acid), antioxidation (e.g., L- pyroglutamic acid and 1-palmitoyl-2-myristoyl-sn-glycero-3-phosphocholine), and energy and growth (e.g., dodecanoic acid and 2-deoxyribose 5-phosphate AMP) (Figure 7E–N).

## 4. Discussion

Dmrt1 is a conserved transcriptional regulator essential for male sexual differentiation and testicular development across species ranging from mammals to teleost. Loss of DMRT1 has been shown to disrupt germ cell development and spermatogenesis [16,17]. In zebrafish, *dmrt1* mutants exhibited abnormal testis development and a female-biased sex ratio [18]. Similarly, overexpression of *dmrt1* induced female-to-male sex reversal in Chinese medaka [19]. In tilapia, *dmrt1*^−/−^ mutants developed gonads to ovaries that could not be reversed to testicular morphology even with aromatase inhibitor treatment, indicating its essential role in male maintenance and spermatogenesis [7]. Correspondingly, *dmrt1*^+/−^ mutants in Chinese tongue sole (F0 generation) displayed aberrant testes lacking spermatocytes or spermatids [13]. In this study, we generated *dmrt1^−/−^* ZZ males (F4 generation) carrying a stable 8 bp deletion in the first exon of *dmrt1* gene. The gonads of these *dmrt1*-deficient mutants developed ovarian lamella-like and ovarian cavity-like structures, resembling the gonadal phenotypes observed in *dmrt1* mutants of zebrafish and tilapia. We then cut the deficient gonads into pieces, and performed the artificial reproduction. However, almost no eggs were fertilized by 10 hpf, and all eggs had died and sunk by 24 hpf. Given the malformed morphology and the absence of successful fertilization, we concluded that *dmrt1* deficiency disrupted spermatogenesis, leading to infertility in *dmrt1^−/−^* ZZ males. Notably, aside from testicular defects, *dmrt1* mutation did not cause any pathological changes in other parenchymal organs, including heart, liver, spleen, and kidney. Together with its predominant expression in testis, these results demonstrated that *dmrt1* is a highly specific and critical regulatory gene in the male reproductive system of Chinese tongue sole.

Since *dmrt1* is a key male determining gene, most existing studies have predominantly focused on the gonadal phenotypes and spermatogenesis in *dmrt1* mutants. Chinese tongue sole exhibits pronounced female-biased dimorphism, with adult females growing significantly faster than males and eventually gaining a much larger body size [13]. This raised the question of whether *dmrt1* knockout would alter the growth traits of mutant males—an intriguing issue that had captured our research interest. Compared with wild-type males, *dmrt1^−/−^* ZZ males first exhibited a significant growth advantage in body weight and body length at 12 mph. This coincides with the initial manifestation of growth dimorphism between wild-type females and males. This advantage not only persisted but was further expanded by 15 mph, when *dmrt1^−/−^* ZZ males reached a body size comparable to that of wild-type females, with no significant differences in weight or length. The influence of sex-determining gene mutations on body size has been documented in Drosophila. Loss of the transformer (*tra*) gene reduced body size in female larvae, suggesting the potential regulation of body size through the sex determination pathway [20]. Therefore, the enhanced growth performance in *dmrt1*-deficient male Chinese tongue sole positioned it as a promising model for future exploration on molecular mechanism beneath sexual size dimorphism in teleosts.

The nutritional components in fish meat, such as moisture, fat, amino acids, and fatty acids, serve as important indicators for assessing fish quality. In our study, most of these components in *dmrt1^−/−^* ZZ males showed no significant differences compared to wild-type males and females. Moreover, the obtained data was close to those in the previous studies [21]. These findings indicated that knockout of *dmrt1* would not markedly alter the muscle nutritional composition in Chinese tongue sole. Similar analysis in *bmp6*-mutant crucian carp (*Carassius auratus*) and *runx2b*-mutant blunt snout bream (*Megalobrama amblycephala*) also revealed no significant differences in flesh quality between mutant and wild-type strains [22,23].

To further investigate the impact of *dmrt1* knockout on metabolic profiles, we performed non-targeted metabolomics analysis on *dmrt1* mutants and wild-type Chinese tongue soles. The results indicated several enriched pathways and differential metabolites in the muscle tissues of *dmrt1* mutants, which were associated with energy and growth processes, anti-oxidation, and neurotransmission. Both lysine degradation and carbon/galactose metabolism generate acetyl-CoA, a key intermediate in energy metabolism [24]. Adequate lysine supplementation in feed has been shown to promote growth in Chinese tongue sole [25]. In addition, arginine and proline metabolism plays a crucial role in myoblast proliferation in human skeletal muscle cells [26]. A shift in these pathways in *dmrt1^−/−^* ZZ males might thus affect growth rate and muscle growth. While this association is preliminary due to the statistical approach employed, it highlights lysine degradation as a priority pathway for functional validation in the context of *dmrt1* deficiency. Additionally, metabolites such as 2-deoxyribose-5-phosphate AMP and 1-palmitoyl-2-myristoyl-sn-glycero-3-phosphocholine indicated changes in nucleotide/energy metabolism and membrane phospholipid remodeling [27], which are essential for sustaining growth and muscular contraction. L-pyroglutamic acid is an intermediate of the γ-glutamyl cycle linked to glutathione turnover [28]. Its elevation, together with enrichment of glutathione metabolism and histidine metabolism, might help regulate redox homeostasis and maintain muscle integrity under the anabolic and hormonal changes accompanying sex reversal. Beyond antioxidant capacity and energy supply, the up-regulated metabolites in *dmrt1*^−/−^ ZZ males suggested that sex reversal might involve enhanced neuromuscular signaling and other beneficial properties such as blood pressure reduction and anti-tumor activity. Dopamine, nudifloramide, and glutamic acid are all involved in neurotransmission [29,30,31]; their elevated levels in muscle may reflect altered neuromuscular activity and motor control associated with the endocrine background in *dmrt1*^−/−^ ZZ males. Val-tyr and dodecanoic acid have been reported to help lower blood pressure and support cardiovascular health [32,33]. Moreover, dodecanoic acid exhibits potential anti-tumor effects against reproductive and liver cancer by inducing oxidative damage and inhibiting cancer cells growth [33,34]. However, the functions of these differentially abundant metabolites in the muscle of tongue sole, as well as their roles in mediating the metabolic alterations caused by *dmrt1* deletion, require further experimental validation.

This study is the first to assess the growth performance and fecundity of the F4 generation of *dmrt1*-edited tongue sole, and to compare the differences in muscle nutritional composition, texture structure, and metabolites between genome-edited fish and wild-type fish. Although sampling was randomized and samples were submitted to a third party for testing, a limitation of this study is that the data analysts were not blinded to the group allocation. On the other hand, during the comparative metabolomic analysis, limitations in sample availability led to relatively small and unbalanced sample sizes in certain groups. Consequently, the analysis of differential metabolites in this study is exploratory in nature and requires validation through larger sample sizes and targeted metabolite verification. We will continue to monitor the muscle nutritional composition and metabolite profiles of the F5 generation, while ensuring blinding to group allocation during data analysis. Furthermore, we plan to investigate the behavioral and immunological parameters of the gene-edited fish and conduct comparative analyses with their wild-type counterparts.

## 5. Conclusions

In conclusion, by integrating growth, reproductive, histological, flesh quality, and non-targeted metabolomic analyses, we demonstrate that targeted disruption of *dmrt1* generates a fast-growing but completely sterile all-male Chinese tongue sole strain whose body size and muscle quality closely resemble those of wild-type females. The F4 *dmrt1*^−/−^ ZZ males showed feminized growth trajectories, ovarian-like gonadal structures, and a complete failure to produce functional sperm, while no major abnormalities were detected in other major organs. Their muscle proximate composition, amino acid, and fatty acid profiles, as well as most texture parameters, were comparable to those of wild-type females and generally fell within the ranges reported for conventional stocks, indicating that *dmrt1* editing might not adversely affect the nutritional or sensory quality of the flesh. Metabolomic profiling further revealed differential enrichment of pathways related to energy provision, antioxidant defense, and neuromuscular function. Together, these results suggested *dmrt1*^−/−^ ZZ males as a valuable fast-growing strain for the Chinese tongue sole aquaculture industry. Moreover, this model offers a useful entry point for dissecting the molecular basis of sexual size dimorphism and for systematically evaluating the effects of genome editing in fish across multiple levels—including physiological, genetic, protein, cellular, and metabolic dimensions.

## Data Availability

The raw data obtained from the metabolome sequencing are publicly available at Figshare (https://doi.org/10.6084/m9.figshare.30656981). The raw data of whole-genome resequencing data have been deposited in the NCBI Sequence Read Archive (SRA) under BioProject accession number PRJNA1392763. Other datasets, including growth characters, fertility, muscle texture, and muscle nutritional composition, were presented in this study are included in the article. Further inquiries can be directed to the corresponding author.

## Supplementary Materials

The following supporting information can be downloaded at: https://www.mdpi.com/article/10.3390/biology15010093/s1, Table S1: The alignment of predicted off-target site with the data of whole-genome resequencing. Table S2: The proximate composition of *dmrt1*^−/−^ ZZ males, wild-type males, and wild-type females; Table S3: Amino acids composition in muscles of *dmrt1*^−/−^ ZZ males, wild-type males, and wild-type female; Table S4: Fatty acids composition in muscles of *dmrt1*^−/−^ ZZ males, wild-type males, and wild-type females; Table S5: The OPLS-DA fit criteria; Table S6: Metabolists with significant difference in the positive ion mode between *dmrt1*^−/−^ ZZ males and wild-type females; Table S7: Metabolists with significant difference in the negative ion mode between *dmrt1*^−/−^ ZZ males and wild-type females; Table S8: Metabolists with significant difference in the positive ion mode between *dmrt1*^−/−^ ZZ males and wild-type males; Table S9: Metabolists with significant difference in the negative ion mode between *dmrt1*^−/−^ ZZ males and wild-type males; Figure S1: Off-target analysis in *dmrt1*^−/−^ ZZ males of Chinese tongue sole; Figure S2: PCA of muscle texture; Figure S3: OPLS-DA score plots and OPLS-DA permutation test plot; Figure S4: Raw data of Figure 3A.

## Abbreviations

The following abbreviations are used in this manuscript:

Dmrt1	Doublesex and Mab-3-related transcription factor 1
LHRH	Luteinizing hormone-releasing hormone
DOM	Domperidone
TALEN	Transcription activator-like effector nuclease
GSD	Genetic sex determination
DMS	*Dmrt1*-mutation screening
Hpf	Hour post-fertilization
Mph	Month post-hatch
PBST	Phosphate-buffered saline with Tween 20
HRP	Horseradish peroxidase
ECL	Electrochemiluminescence
PBS	Phosphate-buffered saline
H&E	Hematoxylin and eosin
TPA	Texture profile analysis
VIP	Variable importance for the projection
PCA	Pareto-scaled principal component analysis
OPLS-DA	Orthogonal partial least-squares discriminant analysis
DAM	Differentially accumulated metabolite
KEGG	Kyoto Encyclopedia of Genes and Genomes
SPSS	Statistical Product and Service Solution
ANOVA	One-way analysis of variance
η^2^	Partial eta squared
ΔDmrt1	The truncated Dmrt1 protein
