# Supplementary material for: Evaluation of an Infertile, All-Male ZZ Line Exhibiting Female-like Growth in Chinese Tongue Sole (Cynoglossus semilaevis): Growth Performance, Flesh Quality, and Muscle Metabolome
Source: Biology (Basel). 2026 Jan 1;15(1):93. doi: 10.3390/biology15010093 (PMC12785089; doi:10.3390/biology15010093)
Supplement: Supplementary file 1 [file biology-15-00093-s001.zip › Supplementary figures.pdf]

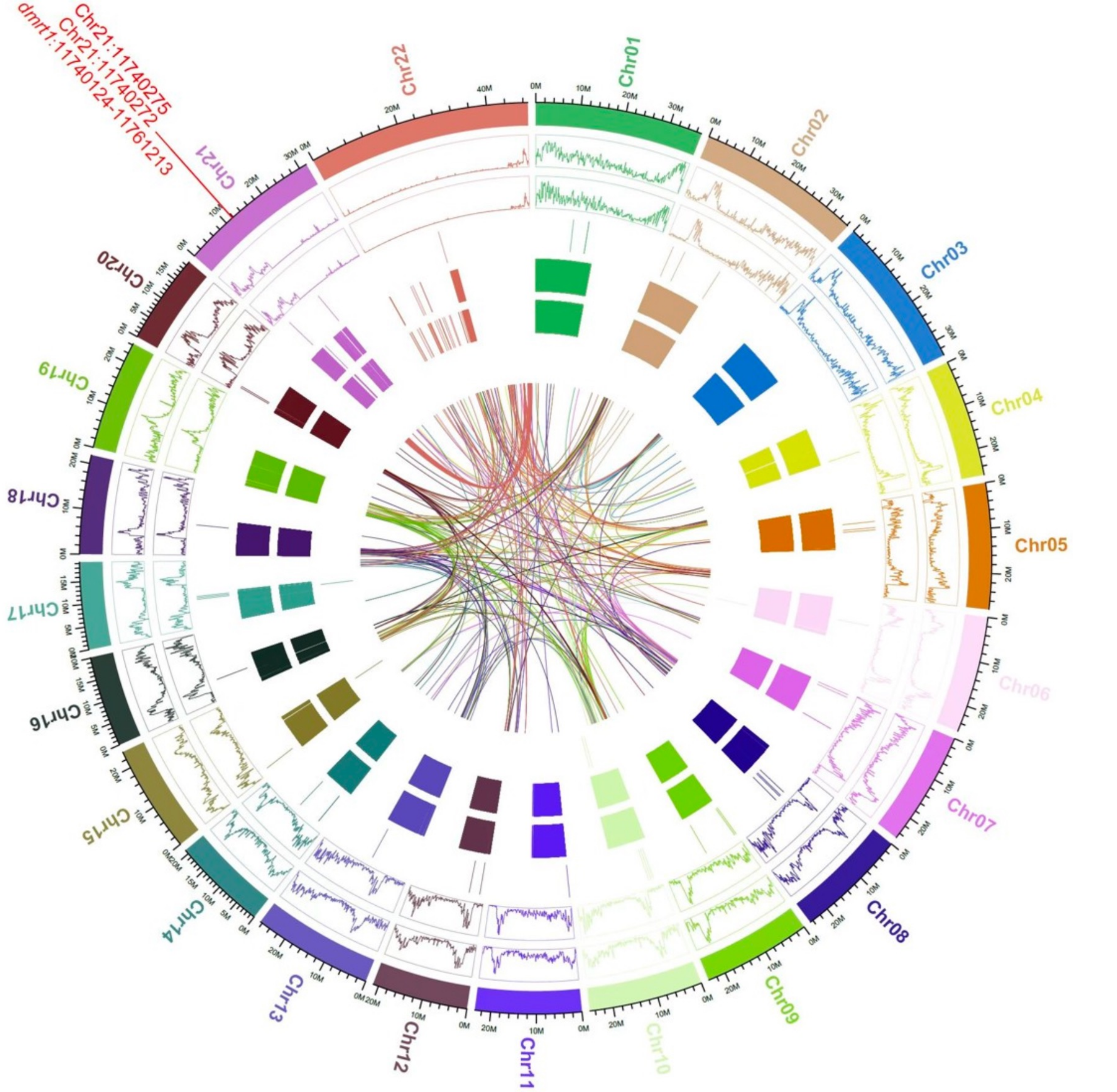

Figure S1. Off-target analysis in *dmrt1*$^{-/-}$ males of Chinese tongue sole. The sequence aligned to the predicted off-target site corresponds to the genomic region of the *dmrt1* gene (located at 11740124-11761213 on the Z chromosome).

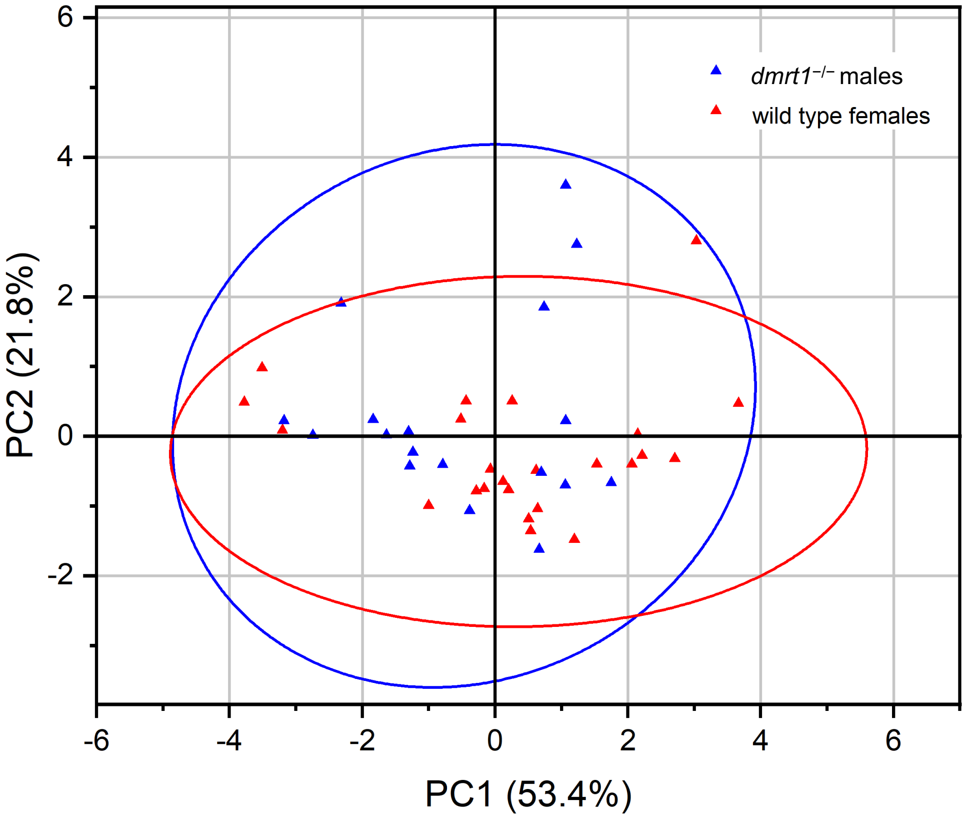

Figure S2. PCA analysis of muscle texture.

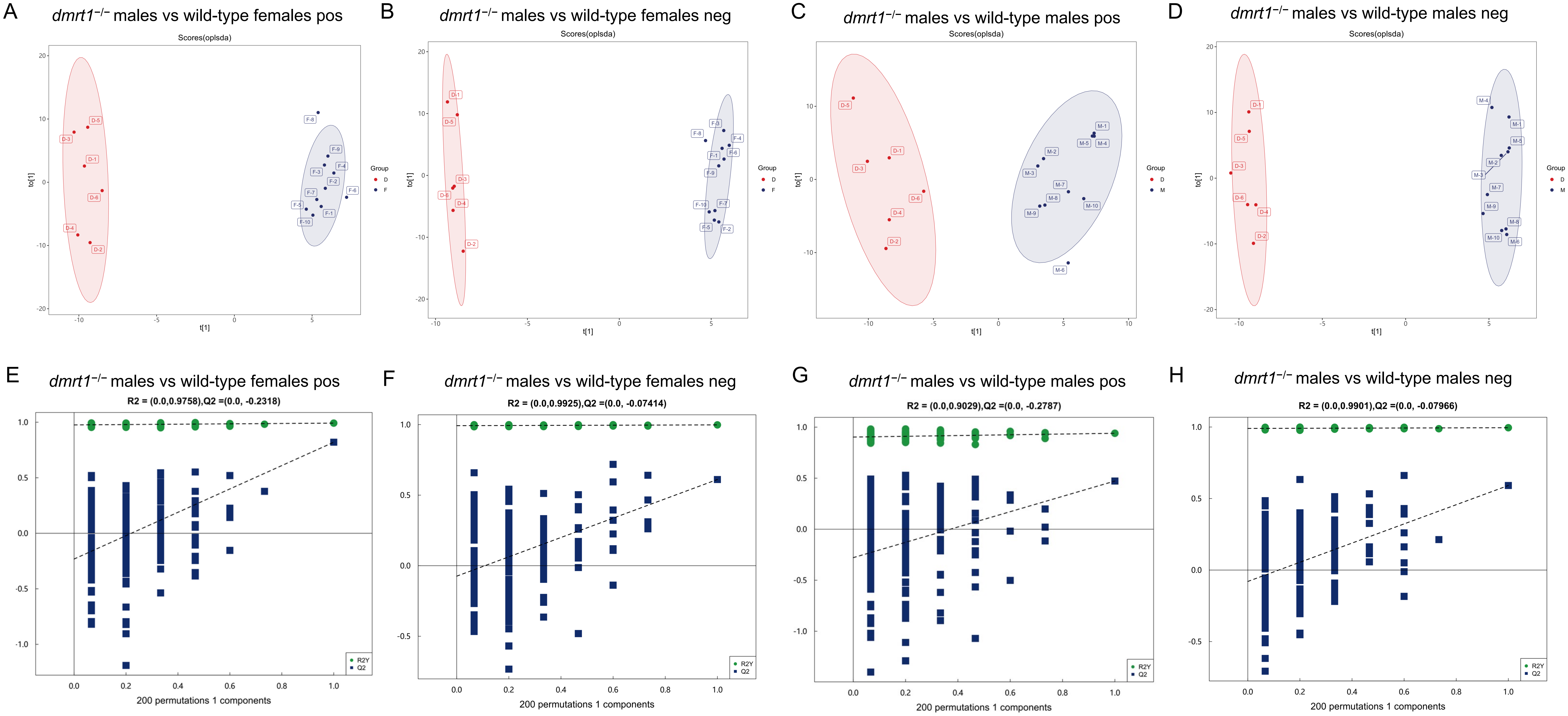

Figure S3. OPLS-DA score plots and OPLS-DA permutation test plot. OPLS-DA score plots between *dmrt1*$^{-/-}$ males and wild-type females in both positive ion mode (A) and negative ion mode (B). OPLS-DA score plots between *dmrt1*$^{-/-}$ males and wild-type females in both negative ion mode (C) and negative ion mode (D). OPLS-DA permutation test plot between *dmrt1*$^{-/-}$ males and wild-type females in both positive ion mode (E) and negative ion mode (F). OPLS-DA permutation test plot between *dmrt1*$^{-/-}$ males males and wild-type females in both negative ion mode (G) and negative ion mode (H).

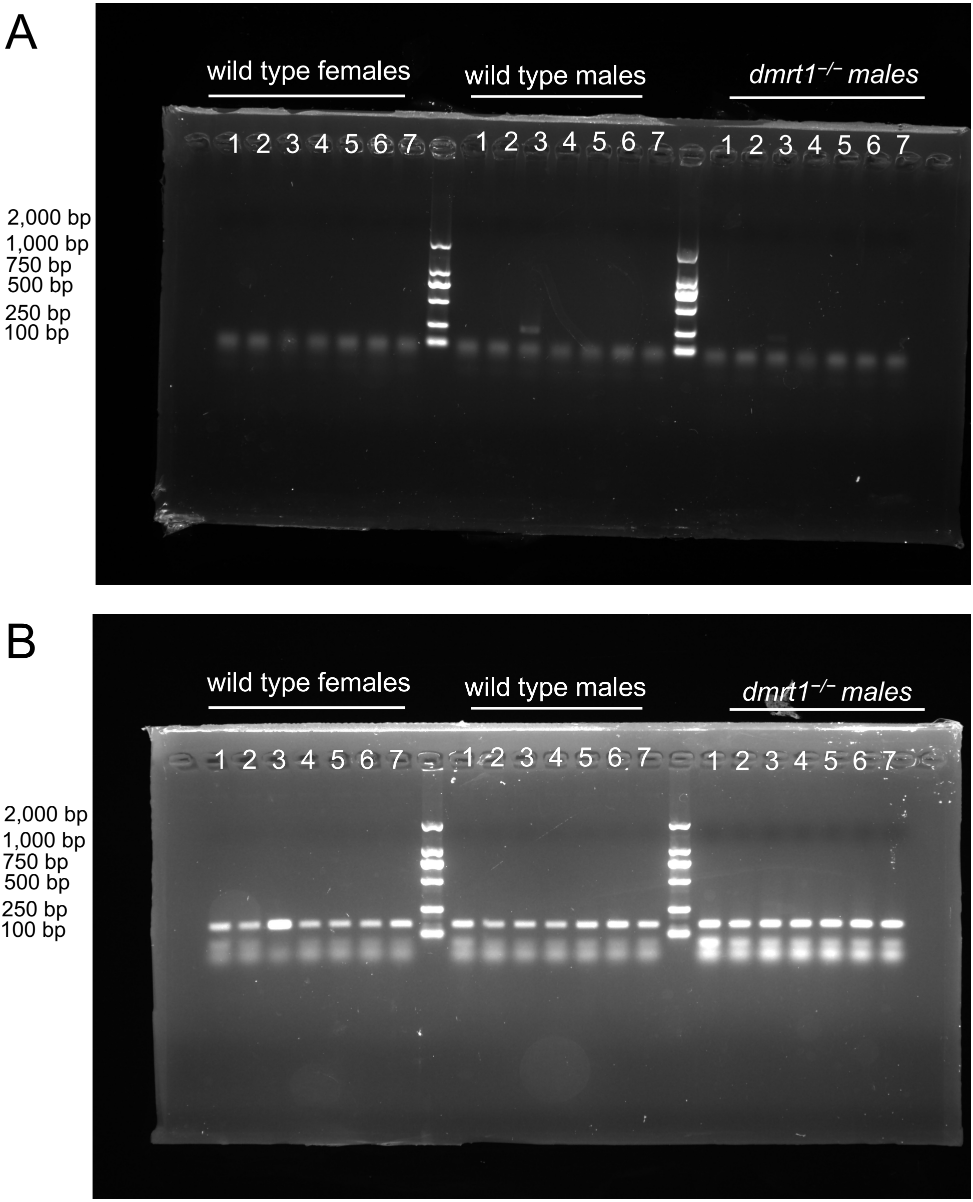

Figure S4. RT-PCR analysis of *dmrt1* (A) in seven tissues of wild type females, wild type males, and *dmrt1*$^{-/-}$ males using *β-actin* as the internal control (B). Tissues included heart (1), liver (2), gonad (3), intestine (4), muscle (5), skin (6), and blood (7).
